# Keeping a track on leptomeningeal disease in non–small cell lung cancer: A single-institution experience with CNSide^TM^

**DOI:** 10.1093/noajnl/vdad150

**Published:** 2023-12-09

**Authors:** Sonam Puri, Rachna Malani, Anna Chalmers, Kathleen Kerrigan, Shiven B Patel, Kelly Monynahan, Laura Cannon, Barbara Blouw, Wallace Akerley

**Affiliations:** Division of Medical Oncology, The Huntsman Cancer Institute at The University of Utah, Salt Lake City, Utah, USA; Division of Medical Oncology, The Huntsman Cancer Institute at The University of Utah, Salt Lake City, Utah, USA; Division of Medical Oncology, The Huntsman Cancer Institute at The University of Utah, Salt Lake City, Utah, USA; Division of Medical Oncology, The Huntsman Cancer Institute at The University of Utah, Salt Lake City, Utah, USA; Division of Medical Oncology, The Huntsman Cancer Institute at The University of Utah, Salt Lake City, Utah, USA; Division of Medical Oncology, The Huntsman Cancer Institute at The University of Utah, Salt Lake City, Utah, USA; Division of Medical Oncology, The Huntsman Cancer Institute at The University of Utah, Salt Lake City, Utah, USA; Biocept, Inc., San Diego, California, USA; Division of Medical Oncology, The Huntsman Cancer Institute at The University of Utah, Salt Lake City, Utah, USA

**Keywords:** cerebrospinal fluid, circulating tumor cells, CNSide, leptomeningeal disease, non–small cell lung cancer

## Abstract

**Background:**

Leptomeningeal disease (LMD) is a devastating complication for patients with advanced cancer. Diagnosis and monitoring the response to therapy remains challenging due to limited sensitivity and specificity of standard-of-care (SOC) diagnostic modalities, including cerebrospinal fluid (CSF) cytology, MRI, and clinical evaluation. These hindrances contribute to the poor survival of LMD patients. CNSide is a CLIA-validated test that detects and characterizes CSF-derived tumor cells and cell-free (cf) DNA. We performed a retrospective analysis on the utility of CNSide to analyze CSF obtained from advanced non–small cell lung cancer (aNSCLC) patients with suspected LMD treated at the Huntsman Cancer Institute in Salt Lake City, UT.

**Methods:**

CNSide was used to evaluate CSF from 15 patients with aNSCLC. CSF tumor cell quantification was performed throughout treatment for 5 patients. CSF tumor cells and cfDNA were characterized for actionable mutations.

**Results:**

In LMD-positive patients, CNSide detected CSF tumor cells in 88% (22/25) samples versus 40% (10/25) for cytology (matched samples). CSF tumor cell numbers tracked response to therapy in 5 patients where CNSide was used to quantify tumor cells throughout treatment. In 75% (9/12) of the patients, genetic alterations were detected in CSF, with the majority representing gene mutations and amplifications with therapeutic potential. The median survival for LMD patients was 16.1 m (5.2-NR).

**Conclusions:**

We show that CNSide can supplement the management of LMD in conjunction with SOC methods for the diagnosis, monitoring response to therapy, and identifying actionable mutations unique to the CSF in patients with LMD.

**Key Points**

- CNSide was used to detect tumor cells and mutations in the cerebrospinal fluid (CSF) of patients with leptomeningeal disease (LMD).
- CNSide had an improved tumor cell detection versus conventional cytology in matched CSF samples.
- Longitudinal changes in CSF tumor cell numbers tracked clinical response in a subset of patients.

**Importance of the Study**

The diagnosis and assessment of response to therapy in patients with leptomeningeal disease (LMD) are challenging due to the limited sensitivity and specificity of standard-of-care diagnostic modalities, including conventional cytology, clinical evaluation, and craniospinal MRI. This contributes to underdiagnosis and poor survival of these patients. CNSide detects and characterizes CSF tumor cells and cell-free DNA. We retrospectively analyzed the use of CNSide in advanced non–small cell lung cancer patients with LMD treated at a single institution and demonstrated an improved tumor cell detection by CNSide compared to cytology in matched CSF samples of LMD-positive patients. Tumor cell numbers assessed throughout treatment appeared to track response to therapy and actionable mutations present in the CSF were identified. An ongoing prospective clinical trial (NCT05414123) will further assess the utility of CNSide in managing LMD and establish the performance characteristics of tumor cell detection versus cytology.

Leptomeningeal disease (LMD) or seeding of tumor cells to the pia and arachnoid mater is a devastating complication in patients with advanced cancer. Approximately 3%–4% with advanced non–small cell lung cancer (aNSCLC) are diagnosed with LMD, and it occurs with a higher frequency in the adenocarcinoma subtype.^1^ The incidence rises to approximately 9% in patients with epidermal growth factor receptor (EGFR)-mutated NSCLC^2^; however, autopsy studies suggest LMD is frequently underdiagnosed.^3,4^ The survival for patients with aNSCLC associated with LMD is dismal, with a median overall survival (OS) of 3–11 months from diagnosis.^1,2,5^

The diagnosis of LMD is often challenging due to the limited sensitivity and specificity of the different diagnostic modalities. Symptoms may range from headaches, confusion, and psychiatric disorders, to cranial nerve deficits, diplopia, limb weakness, and hearing loss.^6^ The diagnostic evaluation includes an MRI of the brain and spine, with or without gadolinium.^1^ MRI may detect leptomeningeal enhancement, hydrocephalus, and subependymal nodules/deposits.^1^; Furthermore, craniospinal MRI results can be interpreted as normal in up to 20% of patients with LMD.^1^ Spinal cord, conus medullaris, and cauda equina involvement may show patchy enhancement of nerve roots and extramedullary nodules. Evaluation of the cerebrospinal fluid (CSF) obtained through a lumbar puncture (LP) is often part of the diagnostic evaluation and may show mild pleocytosis, hypoglycorrhachia, and elevated protein. The opening pressure may also be elevated in 50%–70% of cases. False-negative cytology results are frequent but can be mitigated by performing analysis promptly after CSF has been drawn and using a volume of at least 10 mL.^7^ Positive cytology and suspicious craniospinal MRI findings are enough to make a diagnosis; however, serial LPs are frequently performed to confirm a negative cytology result, especially if there is high clinical suspicion.

Treatment options for patients with aNSCLC and LMD aim to prolong survival and alleviate symptoms.^1,8^ Therapeutic strategies range from whole-brain radiation therapy (WBRT) to intrathecal therapy (IT). More recently, EGFR tyrosine kinase inhibitors (TKIs) have shown potential as a treatment option for patients with EGFRm NSCLC and LMD.^2^ However, treatment options for patients lacking systemic therapy options with intracranial efficacy remain limited. For patients with a low Karnofsky performance status (<60%), with multiple or significant neurological deficits, extensive systemic disease, and bulky central nervous system disease, a palliative/supportive care approach is recommended.^9^ Furthermore, evaluating the CSF for the presence of actionable biomarkers is currently not considered as the standard of care (SOC) in managing LMD.

The design of prospective therapy trials in LMD patients is hampered by limitations in measuring the response to treatment. The Response Assessment in Neuro-Oncology working group has developed a consensus proposal for evaluating patients with LMD; however, the proposed methodology requires further validation in prospective studies.^10^ Different methodologies have been explored, including detecting and quantifying CSF-derived tumor cells (CSF-TCs) using CellSearch, flow cytometry, and CSF-derived cell-free tumor DNA (cfDNA).^11,12^ CellSearch is approved by the Food and Drug Administration for the detection of circulating tumor cells in the blood, and when the platform was adapted to CSF, it was shown to have improved sensitivity and specificity compared to conventional cytology in a prospective study evaluating LMD patients with different epithelial tumors.^13^ Furthermore, serial longitudinal quantitative detection of CSF-TCs was shown to be associated with response to therapy in a prospective study of LMD breast cancer patients treated with IT trastuzumab.^14^ In addition, CSF-TC quantitative assessment at baseline in LMD breast cancer or NSCLC patients treated with proton craniospinal irradiation (pCSI) was shown to be a promising prognostic biomarker of response,^3^ and associated with survival in newly diagnosed LMD patients.^4^ However, the limitations of this platform are that the detection is restricted to cells that express epithelial cell adhesion molecule (EpCAM), and it can detect up to 200 cells per sample.

CNSide (Biocept, San Diego, CA) is a platform that quantifies and characterizes tumor cells and cell-free total nucleic acids (cfTNA) from a single CSF sample. The platform is College of American Pathologists/Clinical Laboratory Improvement Amendments (CAP/CLIA) validated and run commercially at Biocept. Here we describe a retrospective case study series of 15 unique aNSCLC patients treated at the Huntsman Cancer Institute (Salt Lake City, UT) at the University of Utah, where CNSide was used to supplement the SOC procedures to manage their disease.

## Methods

### Patient Population and CSF Collection

We retrospectively evaluated 15 patients with aNSCLC who had suspected, prior known, or a new diagnosis of LMD. All patients were treated at the Huntsman Cancer Institute and had CNSide analysis performed on their CSF at Biocept per the discretion of the treating physician. Two types of samples were used for CNSide analysis: freshly collected samples that were immediately processed for analysis (from 6 patients) and fresh frozen samples (8 patients). For 2 patients, both fresh and frozen samples were used. The frozen samples were collected under an institutional IRB-approved umbrella protocol (89989). The fresh samples were collected at the physician’s discretion as the SOC in managing LMD. Patients agreed to have their data shared per the IRB-approved umbrella protocol (89989). Fresh CSF was obtained from patients that were being treated between April 2020 and October 2022, and frozen samples were collected before CNSide was validated, between June 2017 and January 2020.

Fresh CSF samples were collected per institutional standard procedures, transferred into CEE-Sure CSF collection tubes (Biocept Inc), and sent to Biocept under ambient temperature for CNSide analysis. CNSide is a platform that allows for the detection, enumeration, and biomarker analysis of CSF-TCs, as well as molecular analysis of the supernatant. Frozen samples were generated by collecting fresh CSF per institutional procedures followed by immediate freezing at −80°C followed by dry-ice shipping to Biocept. Fresh and frozen samples were analyzed for molecular biomarker expression using Next-Gen Sequencing (NGS) and Switch Blocker analysis, and fresh samples were used for CSF-TC detection. For fresh samples, the CSF-TC and molecular analysis of the supernatant were performed on the same sample. The CSF-TC detection is CLIA validated and used as a commercial assay at Biocept’s CAP-accredited and CLIA-licensed laboratory in San Diego at the physician’s discretion. Conventional cytology was performed at the institution’s pathology laboratory.

### CSF Tumor Cell Capture and Detection

Fresh CSF samples were centrifuged, and the CSF-TCs pellet was used for CNSide tumor cell enumeration. In summary, cells are captured in the CSF via hybridization with a 10-antibody, followed by several wash steps and incubation with biotinylated secondary antibodies. After several wash steps, biotinylated cells are floated in a streptavidin-coated microfluidic device, resulting in the immobilization of cells. Tumor cells are identified by specific markers via immunocytochemistry analysis, including a mixture of different cytokeratin antibodies as well as CD45 and 4’,6’-diamindino-2-phenylindole (DAPI). Only cells that are positive for tumor-associated cytokeratins and DAPI, as well as negative for CD45, are deemed CSF-TCs and included in the enumeration results. Cells that are DAPI negative or CD45 positive, such as red blood cells and lymphocytes, are excluded from analysis. The method was initially validated for tumor cell detection from the peripheral blood of patients with cancer and is described in more detail by Pecot et al.^15^

### Switch Blocker and NGS Assay

Circulating total nucleic acids (cfTNA) were isolated from the CSF supernatant using the Qiagen viral total nucleic acid kit on the Qiasympohony (Qiagen, Redwood City, CA) and used in Switch Blocker as described in Poole et al.^16^ as well as NGS. For NGS, cfTNA was used to prepare amplicon-based NGS libraries to detect somatic alterations in 12 lung cancer genes using the Oncomine Lung Panel from ThermoFisher. Libraries were templated and sequenced using the Ion Chef and S5 XL Ion Torrent systems. Data were analyzed using Torrent Suite and Ion Reporter software. Reporting and annotation were accomplished using Ion Reporter and Oncomine Knowledgebase software. The volume of CSF required for this analysis is 3 cc.

### FISH Analysis

Cells localized in the microfluidic channels were hybridized with probes detecting amplification for HER2-normalized to CEP17- (Abbott, Des Plaines, IL), cMET-normalized to CEP7- (Biocare Medical, Pancheco, CA), and EGFR-normalized to CEP7- (Oxford Gene Technology, Oxfordshire, UK). Alterations in NTRK (NTRK1 and NTRK3) were detected using the break-apart FISH assay with probes from Oxford Gene Technology, Oxfordshire, UK. CSF-TCs were amplified for HER2 when 1 or more cells showed ≥2.0 HER2 over CEP17 ratio, or≥6 HER2 signals per cell. CSF-TCs were amplified for cMET when 1 or more cells in the CSF displayed ≥2.0 cMET to CEP7 ratio, or ≥5 CMET signals per cell. CSF-TCs were amplified for EGFR when 1 or more cells showed ≥2.0 EGFR to CEP7 ratio, or ≥4 EGFR signals in ≥40% of cells analyzed, or ≥15 EGFR signals in ≥10% of the cells analyzed. The volume of CSF required for the FISH analysis is 2 cc.

## Results

We retrospectively evaluated 15 patients with aNSCLC with suspected or confirmed LMD. All patients were treated at the Huntsman Cancer Institute and had CNSide analysis performed on CSF at different time points at Biocept. A combination of fresh and frozen CSF was used, as described in materials and methods. Frozen samples were analyzed for patients 1–8 as CSF was obtained from these patients prior to CNSide being available. For patients 6 and 7, fresh samples were also tested by CNSide; as for those patients, CSF was collected before and after CNSide was available (see Table 1).

**Table 1.** Patient Demographics and Clinical Characteristics

Patient Number	Sex	Age (Years)	Sample Type	LMD (±)	Clinical Symptoms at the Time of CNSide Analysis	Time from Primary Diagnosis to LMD (Months)	Survival (Days)	Status^*^
1	F	55	Frozen	+	Asymmetric facial numbness	56	515	Expired
2	M	39	Frozen	+	Imbalance, seizures, hydrocephalus	0	155	Expired
3	M	56	Frozen	+	Cranial neuropathies, vision, gait imbalance, urinary incontinence	24	449	Expired
4	M	63	Frozen	+	Imbalance, confusion	9	206	Expired
5	F	43	Frozen	+	Aphasia, saddle anesthesia	56	37	Expired
6	F	63	Frozen & Fresh	+	Aphasia, light sensitivity, gait imbalance	9	1568	Expired
7	M	57	Frozen	+	Gait imbalance	51	1557	Alive
8	F	72	Frozen & Fresh	+	Loss of hearing, gait imbalance	16	1011	Alive
9	F	62	Fresh	+	Vision changes, headaches	0	275	Alive
10	F	59	Fresh	+	Headaches	57	113	Expired
11	M	65	Fresh	+	Confusion, gait imbalance	0	102	Alive
12	M	67	Fresh	+	Diplopia, headaches, dysphagia, facial numbness	0	18	Alive
13	F	67	Fresh	**−**	Facial numbness	NA	NA	Alive
14	F	78	Fresh	**−**	Floaters	NA	NA	Alive
15	F	65	Fresh	**−**	Dysphagia, facial numbness	NA	NA	Alive

Baseline demographics and key clinical characteristics of the 15 unique patients with advanced NSCLC evaluated by CNSide CSF analysis. CSF, cerebrospinal fluid; F, female; LMD, leptomeningeal disease; M, male; yr, years.

^*^Status at time of data analysis.

An LMD-positive diagnosis was defined as meeting at least 2 out of 3 criteria that are commonly used to diagnose LMD: (1) a positive or suspicious craniospinal MRI, (2) a positive or suspicious clinical evaluation, and (3) a cytology result that showed detection of tumor cells.^9^ An LMD-negative diagnosis was defined as a positive or suspicious craniospinal MRI or a suspicious or positive clinical evaluation as described above, but lack of detection of cells by conventional cytology and the absence of LMD-related progression within 12 months. By this definition, 12 patients were LMD positive and 3 were negative. Fresh frozen (from 8 patients) and freshly collected (from 6 patients) CSF samples were analyzed. For 2 patients, both fresh and frozen samples were used (see Table 3).

### Baseline Patient Characteristics

Our aNSCLC cohort’s median age was 63 years, and there was no significant difference in age or gender between LMD-positive and -negative patients (see Table 1). All patients were treated with SOC diagnostics and therapeutic interventions. Treatment consisted of molecularly targeted therapy if an actionable mutation was identified (*n* = 6) or immunotherapy, chemotherapy, whole-brain radiotherapy (WBRT), if no actionable mutation was identified (*n* = 6). Fifty percent LMD-positive patients (*n* = 6) were treated with intrathecal chemotherapy (IT theotepa or methotrexate). The median time between the diagnosis of the primary tumor and the first LMD diagnosis was 12.5 (0–57) months. The median survival for the LMD-positive patients was 16.1 m (5.2—not reached).

### Improved Sensitivity of CSF Tumor Cell Detection With CNSside Compared to Conventional Cytology

Matched fresh CSF samples of LMD-positive patients were used to detect CSF-TCs using conventional cytology and CNSide. Frozen samples were analyzed for tumor cells; however, none were detected as anticipated since the samples were frozen without preservatives. As shown in Table 2, in LMD-positive patients, CNSide detected CSF-TCs in 88% of the samples (22/25), whereas this was 40% (10/25) for conventional cytology. This includes samples that were obtained at diagnosis, as well as longitudinal samples that were obtained from patients during LMD treatment. In addition, for 12 out of 15 cytology-negative samples (80%), CNSide detected tumor cells in the samples. Four patients (9, 10, 11, and 12) had no previous LMD diagnosis. In these patients, CNSide detected tumor cells, in contrast, conventional cytology detected CSF-TCs in 1 out of 4 patients. Five samples were collected from the 3 LMD-negative patients (patients 13, 14, and 15). Neither cytology nor CNSide detected CSF-TCs (Table 2). These patients were deemed negative for LMD as they did not develop progressive symptoms associated with LMD, despite being followed for over a year by serial exams and brain MRIs.

**Table 2.** Comparison of CSF tumor cell capture between CNSide AND Cytology

Patient Number	LMD (±)	Number of CSF Draw	LP/Ommaya (O)	Number of Months Between CSF draws	Cytology (Positive, Negative, Atypical)	CNSide	
						Detected/Not Detected	Cells/mL
6	+	1	O	0	Positive	Detected	15
		2		1.8	Positive	Detected	525
8	+	1	O	0	Negative	Not detected	0
		2		8	Negative	Detected	0.3
		3		27	Negative	Detected	1
		4		30	Negative	Not detected	0
9	+	1*	LP	0	Negative	Detected	13
		2		1.4	Negative	Detected	7
		3		2.7	Negative	Detected	11
		4		6	Negative	Detected	7
		5		21	Negative	Detected	85
		6		22	Negative	Detected	210
10	+	1*	O	0	Negative	Detected	58
		2		1	Positive	Detected	383
		3		2	Positive	Detected	151
		4		2.6	Positive	Detected	514
11	+	1*	O	0	Positive	Detected	19
		2		0.7	Positive	Detected	43
		3		1.4	Positive	Detected	12
		4		2.4	Positive	Detected	7
		5		4	Negative	Detected	5
		6		6	Negative	Detected	5
		7		9	Positive	Detected	17
		8		10.6	Negative	Not detected	0
12	+	1*	LP	NA	Negative	Detected	4
13	**−**	1*	LP	NA	Negative	Not detected	0
14	**−**	1	LP	NA	Negative	Not detected	0
		2		NA	Negative	Not detected	0
		3		NA	Negative	Not detected	0
15	**−**	1*	LP	NA	Negative	Not detected	0

CSF was obtained from 15 unique patients with confirmed LMD at different time points throughout treatment and at diagnosis (indicated by *) as described in Materials and Methods. CSF at each collection was analyzed in parallel for CSF tumor cell presence by CNSide and conventional cytology. For samples analyzed at diagnosis (patients 9, 10, 11, and 12—time point 1, indicated by *), Cytology detected cells in 1 out of 4 CSF samples of LMD-positive patients. In contrast, CNSide detected tumor cells in all 4 samples. Overall, CNSide detected cells in 88% (22/25) of the samples and cytology in 40% (10/25) of the samples. For 3 patients that did not have confirmed LMD (patients 16, 17, and 18), neither CNSide nor cytology detected tumor cells in the CSF. CSF, cerebrospinal fluid; LMD, leptomeningeal disease; LP, lumbar puncture; NA, not applicable.

### Utilization of CNSide to Supplement the Diagnosis and Monitoring of LMD Patients: Case-Based Discussion

In our cohort of patients with aNSCLC, CNSide was used for the diagnosis and monitoring of response to therapy. Here we describe 2 groups of LMD-positive patients where CNSide was integrated to SOC management of disease. Patients in group 1 were diagnosed with LMD prior to CNSide being a commercially available test. In this group, CNSide was used for longitudinal assessment of CSF-TCs to determine the response to therapy. Patients in group 2 did not have a previous LMD diagnosis, and CNSide was used to help with diagnosing LMD, determining actionable biomarkers in the CSF, and to measure response to therapy. Changes in CSF-TC number appeared to track the course of disease and response to treatment for both groups (Table 2).

Group 1 consists of patients 6 and 8. Patient 6 (Table 2) was a 65-year-old female, never smoker, who was diagnosed with aNSCLC with metastasis to the bone, optic nerve, and liver. Plasma NGS showed an EGFR A547T mutation, and she received first-line carboplatin and pemetrexed chemotherapy. The patient subsequently progressed and was diagnosed with LMD a year after her initial diagnosis (conventional cytology positive, MRI brain imaging positive for leptomeningeal enhancement, and acute development of new neurological symptoms). She was treated with WBRT with the placement of an Ommaya port to monitor treatment response. For the next 7 months, the patient was treated with second-line EGFR TKI erlotinib and eventually developed an EGFR T790M mutation, which was detected in the CSF, and treatment was switched to osimertinib. The patient responded well for the subsequent 3 years but eventually experienced symptomatic progression. This did not correspond to any radiological change in the brain MRI. At that time, CNSide had completed CLIA validation and was used for CSF analysis at the last 2 subsequent time points (over nearly 2 months). CNSide showed 15 cells/mL at the first CSF draw, followed by a 35-fold increase to 525 cells/mL. The patient’s LMD continuously progressed, and the patient eventually expired.

Patient 8 (Table 2) is a 72-year-old female, never smoker, diagnosed with aNSCLC with mets to the lung and brain (parenchyma). Limited tumor molecular testing at the time of diagnosis revealed an ALK—ELM4 fusion by IHC. She started first-line therapy with ALK TKI alectinib and was switched to ceritinib for concern of alectinib-induced pneumonitis. Seventeen months after her initial diagnosis, she presented with symptoms suspicious for LMD—acute onset deafness and gait imbalance with MRI brain suggestive of leptomeningeal spread. CSF was negative for tumor cells by cytology (2 independent CSF draws were analyzed; CNSide was unavailable at that time). Based on the imaging and clinical symptoms, the patient was diagnosed with LMD. Treatment was switched to ALK TKI lorlatinib. The patient’s LMD symptoms improved. Serial CSF-TC assessments during the following 2.5 years by CNSide and cytology on matched CSF on lorlatinib showed negative cytology and low/stable cells on CNSide (Table 2).

**Figure 1.**
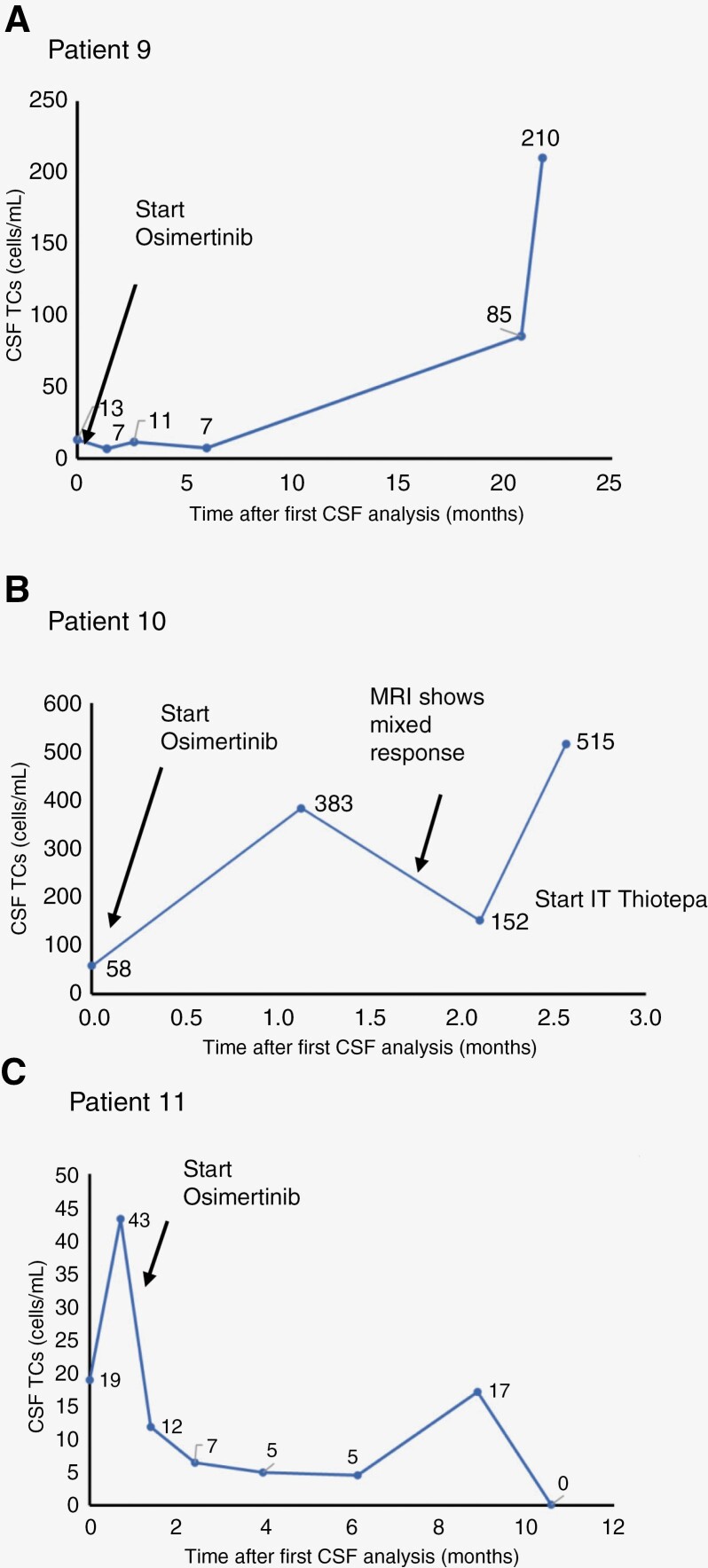
CSF tumor cell number throughout treatment tracks clinical response. CSF was analyzed throughout treatment of patients 9 and 10 at different time intervals. Tumor cells were normalized to volume of CSF. (A) Patient 9 was treated with osimertinib shortly after the first CSF analysis, which led to a decrease in CSF tumor cell number. Cell numbers remained low until nearly 2 years after the first analysis, and further increased rapidly when the patient clinically progressed and declined further treatment. (B) Patient 10 was treated with osimertinib shortly after the first CSF analysis and tumor cell number increased within a month. The MRI showed a mixed response and the patient switched treatment with intrathecal (IT) thiotepa, followed by an increase in CSF tumor cells and clinical progression. (C) Patient 11 was treated with osimertinib after the first CSF analysis, and clinically improved which was paralleled by an overall decrease in CSF-TCs. CSF, cerebrospinal fluid; NA, not applicable; NGS, Next-Generation Sequencing, NP, not performed; Pt. No., patient number.

Group 2 consists of patients 9 and 10. Patient 9 (Table 2, Figure 1) is a 62-year-old female, never smoker, initially diagnosed with early-stage (Stage IA) NSCLC, treated with surgical resection but ultimately developed recurrent metastatic disease with LMD 4 years after her initial diagnosis. LMD symptoms included vision changes, headaches, and facial numbness. The MRI brain was supportive of LMD diagnosis, and CSF was analyzed for cytology as well as CNSide. At diagnosis, CNSide detected tumor cells; however, cytology did not. Tumor molecular testing demonstrated EGFR mutations L833V and H835L, and the patient was treated with EGFR TKI osimertinib. Throughout treatment, cytology remained negative, whereas CNSide demonstrated tumor cell counts that appeared to follow the clinical response, demonstrating a 46% reduction of CSF tumor cells over 6 months (from 13 cells/mL at the first CSF draw to 7 cells/mL at the fourth draw). During this time, clinical LMD symptoms resolved and remained stable. The patient remained on osimertinib therapy for the ensuing year, and CSF was not analyzed. However, symptoms worsened 20 months after LMD diagnosis, and monthly CSF analysis resumed, showing a 12-fold increase in CSF tumor cells compared to the time of diagnosis (from 7 cells/mL at diagnosis to 85 cells/mL at 21 months). The following month, symptoms worsened further, corresponding with an additional 2.5-fold increase of CSF-TCs to 210 cells/mL (Table 2 and Figure 1). NGS, Switch Blocker, and FISH were performed at different time points throughout treatment and revealed mutations in p53 (C242F) and EGFR (H835L) by NGS as well as cMET amplification on the CSF-TCs (Table 3).

**Table 3.** Detection of actionable biomarkers in the CSF by CNSide

Pt. No.	Number of CSF Draw	Fresh/Frozen	Number of Months Between CSF Draws	Molecular Alterations Detected in Primary Tumor Tissue or Blood	CNSide		
					NGS	Switch Blocker	FISH (Number Amplified Cells)
1	1	Frozen	0	EGFR (Exon 19 Del)	None Detected	NP	NP
2	1	Frozen	0	EGFR (Exon 20 insertion V769_D770insASV))	EGFR (M766Q)	NP	NP
	2		1.5		EGFR (M766Q)	NP	NP
3	1	Frozen	0	BRAF (V600E), FANCE rearrangement exon 2 TP53 R175H-subclonal, R282W	BRAF(V600E), PIK3CA(E545K), TP53(R280K)	NP	NP
4	1	Frozen	0	KEAP1 loss, TP53 Q144, RB1splice site 2489 + 1G > T	NRAS (Q61R)	NP	NP
	2		0.5		NRAS (Q61R), KRAS (Q61H)	NP	NP
5	1	Frozen	0	ALK- ELM4 rearrangement	ALK (G1269A), ALK (E1210K)	NP	NP
	2		0.5		ALK (G1269A), ALK (E1210K), PIK3CA(H1047R)	NP	NP
6	1	Frozen	0	EGFR (A547)	EGFR (Del19, M766Q)	NP	NP
	2		3		EGFR (T790M, Del19)	NP	NP
	3		5		EGFR (T790M, Del19)	NP	NP
	5	Fresh	46		NP	EGFR (Del19)	HER2 (2), cMET(6), NTRK1(13)
	6		48		NP	EGFR (Del19)	cMET(1)***
7	1	Frozen	0	ALK- ELM4 rearrangement	None Detected	NP	NP
8	1	Frozen	0	ALK- ELM4 rearrangement	None Detected	NP	NP
	2		8		None Detected	NP	NP
	3	Fresh	27		NP	None detected	NP
	4		30		NP	None detected	NP
9	1	Fresh	0	EGFR (L833V, H835L), TP53 C242F, RB1 splice site 2326-1G > A	NP	None detected	cMET(9)
	2		1.4		NP	None detected	None Detected *
	3		2.7		NP	None detected	cMET (2)
	4		6		NP	None detected	None Detected
	5		21		TP53(C242F), EGFR (H835L)	NP	None Detected
	6		22		NP	None detected	EGFR (30)
10	1	Fresh	0	EGFR (NGS blood) and PD-L1 (blood)	EGFR (L858R, T790M)	NP	None detected
	2		1		NP	L858R	cMET (7)
	3		2		NP	L858R	cMET (21)
	4		2.6		NP	NP	cMET (26)
11	1	Fresh	0	EGFR (L858R) on NGS blood	NP	EGFR (L858R)	None detected
	2		0.7		NP	EGFR (L858R)	None detected
	3		1.4		NP	EGFR (L858R)	EGFR (37)
	4**		2.4		NP	EGFR (L858R)	cMET (9)
	5**		4		NP	EGFR (L858R)	cMET (12), EGFR (13)
	6		6		NP	EGFR (L858R)	cMET (11), EGFR (2)
	7		9		NP	EGFR (L858R)	cMET (69), EGFR (66)
	8		10.6		NP	EGFR (L858R)	NP
12	1	Fresh	NA	NP	KRAS (G12D)	None detected	None detected

CSF was analyzed by CNSide by NGS, Switch Blocker, FISH, and Immuno Cytochemistry per Physician’s choice. Primary tumor tissue and peripheral blood were analyzed per standard of care. Tumor tissue, blood, and CSF were not analyzed at the same time on matched samples. For 75% (9/12) patients, genetic alterations at some point during their treatment were detected in the CSF using either NGS, Switch Blocker technology, or FISH analysis. NP, not performed.

^*^Other alterations were detected, but no gene amplification was observed.

^**^CSF-TCs expressed PD-L1.

^***^HER2 FISH was not performed, and no amplification for NTRK1 was observed.

Patient 10 (Table 2, Figure 1) was a 59-year-old female, never smoker, diagnosed with aNSCLC with malignant pleural effusion. Limited molecular testing at the initial diagnosis was negative for EGFR or ALK mutations. The patient received first-line therapy with dual checkpoint inhibitors on a clinical trial and progressed. Plasma NGS at the time of progression showed an EGFR L858R mutation. The patient received second-line EGFR TKI erlotinib. At progression, an EGFR T790M mutation was found and treated with third-line Osimertinib, followed by fourth-line carboplatin, pemetrexed, and pembrolizumab. Five years after her initial diagnosis, she developed headaches with MRI brain imaging suspicious for LMD. At this time while the initial CSF cytology did not detect malignant cells, CNSide detected 58 cells/mL (Table 2 and Figure 1). NGS analysis of the CSF demonstrated the presence of EGFR mutations L858R and T790M, respectively (see Table 3), and the patient was retreated with Osimertinib. During the ensuing 3 months, CSF-TC numbers initially fluctuated but overall demonstrated an increased cell number of nearly ninefold to 514 cells/mL (last time point at 2.6 months after LMD diagnosis). Between the second and third CSF analyses, CSF-TC numbers decreased by 60% from 383 to 152 cells/mL. In parallel, an MRI brain performed during that time showed a mixed response, and clinical symptoms remained unchanged (cytology remained positive throughout). Treatment was switched to Thiotepa. At a subsequent draw, a 3.4-fold increase in CSF-TC numbers was observed to 514 cells/mL (fourth time point), and the patient continued to progress symptomatically. The Ommaya reservoir was then replaced by a shunt, which provided clinical LMD symptom relief. However, a month later, the patient died in the setting of LMD disease progression.

### Utilization of CNSide for Evaluation of Potentially Targetable Mutations in the CSF

In addition to tracking the course of the disease by CSF-TC analysis, CNSide was also used to evaluate for potentially actionable genomic alterations in CSF by NGS, Switch Blocker, and FISH (see Table 3 for results). Primary tumor tissue and/or peripheral blood was analyzed for molecular alterations per SOC, albeit at a different time point from when the CSF was drawn, and this did not allow for a direct comparison between genetic alterations observed in CSF versus peripheral blood/tissue.

Genetic alterations in the CSF were detected at some point during treatment for 75% (9/12) of LMD-positive patients. Several of these genetic alterations are considered actionable and/or associated with mechanisms of resistance against targeted therapies, such as the ALK (G1269A) mutation,^17^ EGFR (M766Q) mutation,^18^ PIK3CA E545K mutation,^19^ KRAS G12D mutation,^20^ cMET amplification,^21^ and HER2 amplification.^22^ However, there was no modification in systemic therapy to target the specific mutations detected in the CSF in this patient cohort due to the limited data available on the efficacy of these therapies in patients with LMD at the time of CSF analysis.

## Discussion

LMD is a devastating complication of aNSCLC that occurs in 3%–9% of the patients.^2^ It is often underdiagnosed, especially in patients who develop LMD later in their disease course, have progressed on many therapies or have worsening performance status.^3^ Challenges in managing LMD range from adequate diagnosis to lack of effective therapy options, and an inability to adequately assess response to therapy.^5^ This possibly contributes to (1) different treatment modalities for LMD patients across the United States after diagnosis, ranging from hospice recommendations to targeted interventional therapies, and (2) a lack of widespread adoption of CSF evaluation for actionable mutations. Recently, quantifying circulating tumor cells in the CSF using CellSearch showed promising results as a quantitative measure of LMD treatment response to pCSI.^3^ In addition, HER2-positive LMD breast cancer patients demonstrated an improved OS of 10 months when treated with intrathecal anti-HER2-targeted therapy.^23^ These data highlight the feasibility and utility of using CSF analysis to evaluate targetable mutations and quantifying circulating CSF tumor cells to measure therapy response in patients with aNSCLC. CNSide is a proprietary CLIA-validated test commercially run at Biocept as a Laboratory Developed Test and used for CSF analysis in patients with suspected LMD at physician’s discretion. In this case series, we show that in matched CSF samples, CNSide demonstrated an improved tumor cell detection compared to conventional cytology. In addition, CNSide identified cells at LMD diagnosis in 4 patients, whereas the matched conventional CSF cytology assessments were negative. In addition, we demonstrate tumor cell numbers assessed at different time intervals in a subset of patients where CSF was analyzed throughout treatment appear to track the clinical response to therapy.

Furthermore, molecular analysis of the CSF demonstrated the presence of genetic alterations that are potentially targetable or associated with mechanisms of resistance to therapies targeting EGFR or ALK, such as EGFR (M766Q), KRASG12D, ALK (G1269A), PIK3CA E545K mutations, and amplification in cMET and HER2.^17–21^ While at the time of CSF analysis, there was limited clinical trial data available on the safety and efficacy of drugs targeting these specific mutations in NSCLC patients with LMD, over the years, several drugs have been developed that demonstrate clinical benefit for patients with brain metastases, including LMD. A case study of 6 HER2-amplified LMD patients treated with trastuzumab-deruxtecan (T-Dx) showed a median OS of 12.5 months.^24^ Recently, T-Dx was approved for HER2-mutation-positive NSCLC patients.^25^ Additionally, osimertinib (160 mg/day) has shown benefit in EGFR-mutated NSCLC patients with LMD (Phase I of the BLOOM study) with a median progression-free survival of 8.6 months and median OS of 11 months.^2^ A prospective study (FORESEE Study | NCT05414123) is ongoing that will further assess the utility of the assay in managing patients with LMD and establish the performance characteristics of tumor cell detection compared to cytology.
